# Personality Traits and Sociodemographic Factors Associated with the Use of E-Cigarettes, Waterpipe and Conventional Cigarettes among Medical University Students

**DOI:** 10.3390/ijerph19127000

**Published:** 2022-06-08

**Authors:** Yap Yew Shen, Nurul Hanis Ramzi, Divya Gopinath

**Affiliations:** 1 School of Medicine, International Medical University, Kuala Lumpur 57000, Malaysia; yap.yewshen@student.imu.edu.my; 2 Institute for Research, Development & Innovation, International Medical University, Kuala Lumpur 57000, Malaysia; nurulhanis@imu.edu.my; 3 Clinical Oral Health Sciences, School of Dentistry, International Medical University, Kuala Lumpur 57000, Malaysia

**Keywords:** tobacco use, cigarettes, e-cigarettes, water pipe, personality model

## Abstract

This study aimed to investigate the prevalence and sociodemographic characteristics of smokers in a private medical university in Malaysia and to examine whether there is an association between personality traits and various smoking types. There were 468 participants in this study and the mean age was 20.97 years (±2.743). The prevalence of conventical cigarette users, e-cigarette users, and water pipe users was 4.7%, 6.4%, and 4.0%, respectively. Parents’ annual income (*p* = 0.001) and ethnicity (*p* < 0.001) were significantly associated with the current smoker group. Binary logistic regression modelling revealed that study participants with either Malay (OR 4.23, 95% CI 1.73, 10.34) or Chinese (OR 4.16, 95% CI 1.98, 8.73) ethnicity were approximately four times more likely to use tobacco products compared with study participants with Indian and Other ethnicities. Lower parents’ annual income was almost four times more likely to be associated with smoking behaviour (OR 3.82, 95% CI 1.58, 9.27). Significant differences in mean personality traits score of Openness (*p* = 0.018) and Extraversion (*p* = 0.004) were observed between never-smoker and current smoker study participants. In addition, cigarette users scored higher in Conscientiousness personality traits compared with non-cigarette whereas e-cigarette users and waterpipe users scored lower in Extraversion (*p* = 0.02). Post-hoc analysis revealed that the never-smoker group scored higher in Extraversion compared with the dual tobacco user group (*p* = 0.03). In addition, the single tobacco user group scored higher in Agreeableness personality trait compared with the never-smoker group (*p* = 0.01). Deeper understanding of the different cognitive dimensions, ethnicities, and educational backgrounds can potentially impact smoking prevention and cessation programs.

## 1. Introduction

The global tobacco epidemic, a scourge that has wreaked havoc for countless years, remains till this day as the single largest cause of preventable death worldwide, causing 8.7 million deaths yearly according to the World Health Organization [1]. Though great strides have been achieved in curbing smoking, such as the implementation of the WHO Framework Convention of Tobacco Control in 181 countries, the end of tobacco is still a distant goal, with the constant increase in global population masking the harsh reality that the number of smokers has increased despite global smoking prevalence rates being shown to have decreased [2,3]. The rise in popularity of e-cigarettes must also not be ignored, with an increased prevalence of e-cigarette use being reported in many countries, as well as a boom in global sales of electronic nicotine delivery devices (ENDS), valued at US$15 billion in 2019, more than quintupling the prior sales of 2014 which sat at US$2.76 billion [1,4,5]. Electronic cigarettes being in vogue also poses a threat to the tobacco endgame, with research showing that youths that use e-cigarettes are significantly more likely to initiate conventional cigarette smoking than those who have never used e-cigarettes, with one study reporting that e-cigarette users had up to 6.17 times the odds of initiating conventional cigarette smoking compared with people that never used e-cigarettes [1,6,7]. Youths have been particularly attracted to electronic cigarettes due to the various compelling flavours being offered on the market as well as the smell of e-cigarette smoke being relatively more pleasant compared with conventional cigarettes [1,8]. The popularity of e-cigarette-based media on various social networking platforms that positively portray e-cigarette smoking is also an attractive method targeted at the youth as well as the sentiment that e-cigarettes are less harmful than conventional cigarette smoking, although research has shown that e-cigarettes have elicited similar inflammatory responses to regular smoking [9]. Though the long-term effects of e-cigarette use are still unclear, there are still many parallels that can be drawn between e-cigarette smoking and traditional cigarette smoking. Water pipe smoking, also known as shisha and hookah, remains popular among the youth today and continues to serve as an obstacle that impedes the progress toward the tobacco control target [10]. Studies have shown that waterpipe smoking has increased the chances of cigarette smoking initiation, further hampering the eradication of tobacco as well as endangering the health of youths [11,12,13]. Many of the youth who continue to smoke water pipes suffer from a lack of knowledge and misconceptions about the actual health impacts of shisha smoking, with many believing it to be less harmful and addictive than conventional cigarette smoking [14]. Water pipe smoking might even be considered as more harmful than conventional cigarette smoking due to shisha smoking sessions generally lasting longer than conventional cigarette smoking, leading to increased smoke inhalation [15].

Personality traits are long-lasting characteristics and important predictors of behaviour [16]. Personality traits have proven to be linked to both health-promoting practices, as well as health risk behaviours [17,18,19]. The Five-Factor Model of Personality, often referred to as OCEAN, encompasses five traits which are Openness (O), Conscientiousness (C), Extraversion (E), Agreeableness (A), and Neuroticism (N), which are made up of other unique, specific personality aspects known as facets [20]. The traits within the model have shown to be heritable as well as generalisable across cultures, as well as have been studied and linked to various health behaviours, including smoking [16,20]. Openness (O) represents the tendency of an individual to have a broader range of interests, higher sensitivity towards art and beauty, as well as to seek out new experiences. Conscientious (C) individuals lean towards being objective focused, careful, and organised. Extraverted (E) individuals tend to be more assertive, sociable, and talkative in social situations. Agreeableness (A) encompasses individuals who tend to cooperate, maintain peaceful harmonious relationships with others, as well as empathise. Neuroticism (N) in individuals refers to their tendency to experience negative emotions and moods, such as anxiety, depression, and emotional volatility [20]. Studies have shown that prevention programmes that utilise personality traits in targeting high-risk individuals have proven to be effective in the early prevention of substance use [21,22]. These findings highlight the importance of personality-targeted prevention programmes for the various kinds of smoking for intervention to be adequately carried out promptly, such as for high-risk adolescents who otherwise would not have had access to such prevention programmes.

In Malaysia, although many sociodemographic studies on the issue of smoking have already been carried out, there have been none of which pertain to personality traits influencing smoking habits. However, there have been studies globally that have linked personality traits to conventional cigarette smoking. One study found that smokers had higher Neuroticism, lower Agreeableness, and lower Conscientiousness compared with non-smokers, while Openness and Extraversion did not show any significant differences [16]. However, a meta-analysis reported that current smoking was associated with high Extraversion, alongside high Neuroticism and low Conscientiousness, with Agreeableness not contributing towards being a current smoker [23]. Cultural differences across countries being a possible factor for the differences in personality traits of conventional cigarette smokers was also highlighted by another meta-analysis. Moreover, whether there is any impact of professional attributes of young adults and smoking types have not been comprehensively studied yet. Thus, our study aimed to explore the factors associated with tobacco usage, to create tobacco users’ profiles based on sociodemographic distribution, and personality domain features, in a private Malaysian medical university consisting exclusively of health care students who would be future healthcare professionals.

## 2. Materials and Methods

### 2.1. Settings and Study Design

A cross-sectional study was conducted at International Medical University (IMU), KualaLumpur, Malaysia between August and October 2021 with a population of current full-time students. The medical university is exclusively for medical, dental, pharmacy, nursing, and several other allied health care courses and hence our study population consisted of healthcare students. An online survey questionnaire was given to undergraduate and postgraduate student cohorts via e-mail and Microsoft Teams platform. Based on the estimated student population of approximately 3000 students, the minimum sample size required to achieve a 95% confidence level with a 5% margin of error was N = 340 [24,25]. The sample size was derived through Cochran’s sample size formula. The eligibility criteria were to include all full-time students above the age of 18 years old. All participants signed an informed consent form to permit information obtained through questionnaires to be used. Participants who did not consent to the study and did not complete the questionnaire were excluded from the study. The study was independently reviewed and approved by the Institutional Joint Committee for Ethics and Research, International Medical University (BMS I/2020(28)).

### 2.2. Smoking Behaviour Assessment

The Global Adult Tobacco Survey (GATS) serves as a global standard for systematically monitoring adult tobacco use and tracking key tobacco control indicators [26]. Our questionnaire was used to record the respondents’ sociodemographic data, smoking history, and status and among others was adopted and modified from the GATS tool. The sociodemographic section covered sex, age, parents’ annual income, ethnicity, and educational level. Parents’ annual income was categorised as, ‘>RM100,000′, ‘RM100,000−RM300,000′, and ‘>RM300,000′. The educational status of the participant was either ‘undergraduate’ or ‘postgraduate’. Ethnicity was grouped as ‘Malay’, ‘Chinese’, ‘Malaysian Indian’, and ‘International’.

Participants were asked if they had ever smoked a cigarette, e-cigarette, or water pipe, their parent’s smoking status, age at smoking initiation, amount of smoking, and type of cigarette used. The current smoker category was for the respondents who answered ‘Daily’ or ‘Occasionally’ and those who answered with ‘Not at all’ were categorised as never-smoker status. The current smoker status category was then stratified further into two groups, ‘Single Tobacco Users’ if the respondents had answered ‘Daily’ or ‘Occasionally’ to only one smoking category and ‘Multiple Tobacco Users’ if the respondents answered as such to more than one smoking category.

### 2.3. Personality Traits Assessment: OCEAN

The Big Five OCEAN (Openness, Conscientiousness, Extraversion, Agreeableness and Neuroticism) Model was used to evaluate the personality traits of each study participant. The OCEAN questionnaire has been proven to be valid and reliable in Malaysia [27]. The OCEAN questionnaire is a 5-Point Likert scale with 43 items that are based on the five personality constructs of Openness, Conscientiousness, Extraversion, Agreeableness and Neuroticism. The responses were scored with Strongly Disagree (1), Disagree (2), Neutral (3), Agree (4) and Strongly Agree (5). The specifically designated items were then reverse-scored, with 1 changed to 5, 2 to 4, 4 to 2, and 5 to 1. The total score for each personality construct was then calculated. The survey was then pilot tested on 20 students to increase the validity of the content of the measuring instrument.

### 2.4. Statistical Analysis

The data gathered were analysed using IBM SPSS Statistics for Windows, version 26.0 software (IBM Corp., Armonk, NY, USA). The internal reliability of the OCEAN questionnaire was analysed through Cronbach’s alpha and an alpha value of ≥0.7 was deemed to be reliable. Pearson’s chi-squared test (χ^2^) and independent student’s *t*-test were used to comparing the sociodemographic factors (age, sex, ethnicity, parent’s annual income, and education level) and personality constructs (Openness, Conscientiousness, Extraversion, Agreeableness and Neuroticism) between the smoking statuses of study participants. Multivariate analysis of variance (MANOVA) was used to determine the association between the personality traits and smoking status (never-smoker, single, and multiple tobacco product users) of the study participants, adjusted with covariates (sex, ethnicity, and parent’s annual income). In post hoc analysis of never-smoker, single, and multiple tobacco product user groups, the Bonferroni procedure was applied. The logistic regression model was used to estimate the sociodemographic factors which influence the smoking status of the study participants. The dependent and independent variables were the smoking status and sociodemographic of the participant. The odds ratios (OR) along with respective 95% confidence intervals (CIs) were reported. Pearson’s correlation coefficient was used to determine the relationship among the personality constructs. The correlation coefficient ranges from −1 to +1, where a score of ±1 shows a perfect positive or negative linear relationship and a score of zero shows no correlation between the two variables. Finally, a 2-sided 5% significance level was used for all statistical inferences.

## 3. Results

Overall, the prevalence of current smoking among university students was 10.3%, and the mean age of smokers was 21.64 (±3.0) years. The prevalence of cigarette users, e-cigarette users, and water pipe users were 4.7% (n = 22), 6.4% (n = 30) and 4.0% (n = 19), respectively. There was no age and sex difference between smokers and never-smokers in the study (see Table 1). However, there were more males (n = 12, 54.5%) than females (n = 10, 45.5%) who used cigarette products (*p* = 0.008, see Table 2) when compared between cigarette user and non-cigarette user groups.

Parents’ annual income (*p* = 0.001) and ethnicity (*p* < 0.001) were significantly associated with the current smoker group. Binary logistic regression modelling (never-smoker vs. smoker group as dependent variable) revealed that study participants with either Malay (OR 4.23, 95% CI 1.73, 10.34, *p* = 0.002) or Chinese (OR 4.16, 95% CI 1.98, 8.73, *p* < 0.0001) ethnicity were approximately four times more likely to use tobacco products compared with study participants with Indian and Other ethnicities. The OR for the parents’ annual income <RM100,000 was 3.82 with a 95% confidence interval of [1.58, 9.27] (Table 2). This suggests that those with lower parents’ annual income were almost four times more likely to smoke than those who had higher parents’ annual income. However, further analysis by the multinomial logistic regression modelling (never-smoker, single product users and multiple product users as dependent variables) showed that males have lower tendencies of becoming multiple tobacco users with the odds ratio of 0.24 compared with females, and Chinese ethnicity was associated with multiple smoking status with the odds ratio of becoming a multiple tobacco user 0.11 times lower for Chinese compared with Malay and Indian ethnicities (See Table 3).

The personality traits of Extraversion (*p* = 0.02) and Agreeableness (*p* = 0.02) were associated with participants with smoking status (Table 3). Post-hoc analysis revealed that the never-smoker group scored higher in Extraversion by mean differences of 2.99 ± 1.13 compared with the dual tobacco user group (*p* = 0.03). In addition, the single tobacco user group scored higher in the Agreeableness personality trait compared with the never-smoker group by a mean difference of 2.25 ± 0.79 (*p* = 0.01). Significant differences in mean personality traits score of Openness (*p* = 0.018) and Extraversion (*p* = 0.004) were observed between never-smoker and current smoker study participants. In addition, cigarette users scored higher in the Conscientiousness personality trait compared with non-cigarette users by a mean difference of 2.04 ± 1.05 (*p* = 0.03) (Table 4).

All five scales of Openness, Conscientiousness, Extraversion, Agreeableness, and Neuroticism personality constructs had good internal consistency reliabilities with Cronbach alpha ranging from 0.70 to 0.82. Big five personality traits showed non-zero correlations with each other, ranging between −0.11 and 0.72 (all *p* < 0.05) (see Table 5). Among them, openness and conscientiousness had the highest Pearson’s correlation coefficient (r = 0.72, *p* < 0.05). The correlation matrix among the five personality traits indicated that the absolute values of the correlation coefficients were lower than the acceptable cut-off point of 0.8 for inclusion in multiple regression analysis [28].

## 4. Discussion

Understanding the sociodemographic factors and personality traits associated with the use of alternate products among university students can help health professionals in assessing population health risks and designing tailored intervention activities. Through this study, we examined the relationship between sociodemographic factors and exclusive e-cigarette, water pipe, or conventional cigarette use as well as concurrent use of multiple products. We also investigated the association between personality traits and the use of e-cigarettes, water pipes, or conventional cigarettes among university students. To the best of our knowledge, this is the first study to explore the association between personality traits and tobacco use among university students in Malaysia.

The prevalence of conventional cigarette smoking in our university which was 10.3% was significantly lower than the prevalence of smoking found in the previous study of 29% [29]. Water pipe smoking prevalence was 4.0% which was lower compared with the studies conducted in a Malaysian university (30%) and specifically in a medical university (20%) [10,14]. The prevalence of e-cigarette users in our study was 6.4%, which is higher than the prevalence of e-cigarette smoking in Malaysia (4.9%), but lower than the prevalence within the 20–24 year age group (14.7%), which encompasses the majority of the research participants [30]. Such disparity between the prevalence rates may be explained due to the differences in the institutions where the study was carried out. With our institution being a medical university, the better awareness and understanding of students of the negative health effects of smoking may have led to the lower prevalence of various smoking methods as the previous study conducted in a medical university also reflected lower smoking prevalence rates compared with other universities. The student population of our university, which has a higher proportion of female students, may also have contributed to these differences, as numerous previous studies have already concluded that female smoking prevalence is significantly lower compared with that of males for the mentioned smoking methods [10,14,30]. The large difference in water pipe smoking prevalence between our university and the previously studied Malaysian medical university may be explained by the ethnic majority of participants in the two studies. In our study, the majority of participants were Chinese whereas, in the previously conducted study, the majority of participants were Malay [14]. The NHMS 2019, which reported that Malays had a higher prevalence of smoking compared with Chinese, may explain the large difference in water pipe smoking prevalence between the two institutions [30]. It should be noted that e-cigarette prevalence was still the highest among the three methods of smoking that we explored namely conventional, e-cigarettes and waterpipe which may be due to the lack of awareness of its negative health effects as well as the lack of information regarding the long-term use of electronic cigarettes. The higher prevalence of e-cigarette smoking may also be related to the age of the participants, as the mean age of participants was 20.97, which is within the age group of the highest e-cigarette smoking prevalence in NHMS 2019 [30].

There were also differences between the income groups as well; as based on the NHMS, respondents from higher income groups had a significantly lower prevalence of smoking which was due to findings that higher income was a protective factor against cigarette smoking [30]. For e-cigarette smokers, once again those from the highest parents’ annual income group as well as respondents of Malaysian Indian and International ethnicity had significantly higher odds of being e-cigarette smokers. The income group findings were consistent with NHMS findings where the highest prevalence of e-cigarette smoking was observed in the high-income group quintile [30]. The higher upfront prices required for e-cigarette smoking may be an explanation for this finding, making accessibility to e-cigarettes more difficult for those in lower-income groups compared with conventional cigarettes [31]. Another possible explanation is that individuals from higher income groups utilise and consume social media more than the lower-income groups, which may increase their exposure to e-cigarette-based trends on social media [32]. Coupled with the positive portrayal of e-cigarette smoking on social media, this may explain the high prevalence of e-cigarette smoking in higher-income groups [9]. Sex was not found to be significantly associated with e-cigarettes and water pipes, which differs from the previous studies carried out on both smoking methods which reported that males had significantly higher odds of being smokers of both e-cigarettes and water pipes [10,14,30]. Moreover, when we compared the association of sex with single and multiple product users, sex also was found to have a role in the odds of a person being a multiple product user or single user. These results could be attributed to the fact that 75% of the students in our university are females and this is reflected in our sample population as well.

Regarding the association between personality traits and various smoking methods, Conscientiousness was significantly associated with conventional cigarette smoking, which was contrary to the findings of previous studies [16,23,33]. Studies that have shown associations with Conscientiousness have found that smokers generally exhibit low Conscientiousness, which has previously been related to health risk behaviours such as smoking [16,23,33,34]. However, our population consisted of health care students, and it has been shown previously that medical students often score on high conscientiousness with higher self-achievement and self-discipline that significantly predict their professional attributes [35]. This could have reflected in the association between higher consciousness and smoking in this medical university cohort.

The effect of Neuroticism on any kind of smoking was not significant in our study, which is inconsistent with a few previous studies. This could again be attributed to the fact that our cohort consisted of health care students who are more aware about the harmful effects of smoking. Moreover, cultural, economic, and political determinants of smoking could also be considered in the interpretation of differences in research outcomes. Moreover, it is interesting to note that e-cigarette and waterpipe users scored low in Extraversion in comparison with non-users. Previously, several studies have found an association between Extraversion and smoking. According to previous studies, these extraverted individuals may be more susceptible to smoking due to the satisfaction gained from fraternizing amongst peers who share the same smoking habit as well as a reported increased sensitivity towards nicotine in sensation-seeking individuals, hence leading to higher odds of smoking e-cigarettes and water pipes [36]. However, with our study population being healthcare students, the knowledge of the adverse health consequences of tobacco use and stringent social policy against these in Malaysia might have had their influence on these associations [37,38]. Moreover, personality effects can also be influenced by the age group. Those with younger ages, especially university students with lower Extraversion, may possibly have a lower chance to associate with smokers due to their smaller social connections [39]. This may indicate that the less extraverted people are less likely to become smokers. Moreover, it could also be attributed to the fact that the majority of the smokers in our study were females, whereas extraversion has been associated with male smokers [40].

Agreeableness might not have shown any significant associations with e-cigarette and water pipe smoking due to attitudes towards these two smoking methods being mixed. Negative perception toward e-cigarette and waterpipe smoking has not been established as it has been with conventional cigarettes amongst the community [41,42,43]. Hence, because of the lack of concrete opinion within the general public, this might explain why there were no significant associations observed between these smoking methods and Agreeableness, as a previous explanation stated that individuals with low Agreeableness smoked as a sign of rebellious behaviour [16]. The present study has implications for improving our understanding of smoking and smoking cessation programs. Considerations of different cognitive dimensions, ethnicity, and educational backgrounds during designing tobacco cessation programs for university students can increase the likelihood of success after tobacco interventional attempts. A recent study highlighted that genetic determinants could also play a role in determining smoking behaviour [44], which could be the reason why the there is difference in association with personalities and smoking behaviour across populations and cultures.

Several limitations must be addressed with regard to the current study, the first of which is the potential for respondent fatigue [45]. Due to the survey being carried out online, it was not possible to control the environment in which the questionnaire was answered, nor to assess the quality of responses provided by the participants, which may have led to potential errors. In addition, with the low number of smokers obtained from the sample of this study, insufficient representation of these smokers may have occurred, leading to inconsistencies with previous studies such as sex being found to be insignificant for all methods of smoking in this study despite significant associations being observed in other studies. Due to the low number of smokers, some associations between personality traits and smoking may have failed to be elucidated, such as was displayed with conventional cigarette smokers. Lastly, due to the limitations of a single-centre study, our findings may not accurately represent other university populations. This can be improved by conducting a multi-institutional study, which would result in more accurate results.

## 5. Conclusions

Our study has enhanced our understanding of the association of personality with the various types of smoking among medical university students. Due to an association being established, future personality-tailored preventative programmes may be planned and executed to further curb the prevalence of smoking. As we have new evidence on the associations of genetic and environmental factors on tobacco use, future research should examine this paradigm using a behavioural genetics approach.

## Figures and Tables

**Table 1:** Demographic characteristics and personality traits score of study participants.

Subject Characteristics	Never-Smoker	Smoker ^#^	*p*-Value *
	(n = 420)	(n = 48)	
Age (±SD)	20.96 (2.8)	21.64 (3.0)	0.069
Personality Traits (±SD)			
Openness	26.5 (5.1)	24.9 (5.4)	**0.018**
Conscientiousness	24.4 (4.7)	25.2 (5.5)	0.145
Extraversion	22.6 (4.6)	20.7 (4.7)	**0.004**
Agreeableness	21.0 (4.2)	21.9 (5.5)	0.088
Neuroticism	23.0 (4.8)	22.5 (5.4)	0.267
Sex (%)			
Male	117 (27.9)	20 (41.7)	0.046
Female	303 (72.1)	28 (58.3)	
Parents’ Annual Income (%)			
<RM100,000	172 (41)	10 (20.8)	**<0.001**
RM100,000–RM300,000	182 (43.3)	20 (41.7)	
>RM300,000	66 (15.7)	18 (37.5)	
Ethnicity (%)			
Malay	25 (6.0)	2 (4.2)	**<0.001**
Chinese	297 (70.7)	18 (37.5)	
Indian	41 (9.8)	9 (18.8)	
Others	57 (13.6)	19 (39.6)	
Educational Level (%)			
Undergraduate	407 (96.9)	47 (97.9)	0.697
Postgraduate	13 (3.1)	1 (2.1)	

Data are presented as mean (±SD) for continuous (age and personality traits score) variables and as a frequency and percentage for categorical variable. # Smoker group inclusive of cigarette, e-cigarette, or water-pipe/shisha users. * Comparisons between smoking status groups used 2-sided independent *t*-test for continuous variables and χ^2^ test between categorical variables. Significant findings appear in bold.

**Table 2:** Binary logistic regression analysis between sociodemographic factors and smoking status of the study participants (smoker vs. never-smoker).

Variable	OR	95% CI	
		Lower	Upper
**Ethnic (Chinese)**	**4.16**	**1.98**	**8.73**
Ethnic (Indian)	1.24	0.27	5.79
**Ethnic (Malay)**	**4.23**	**1.73**	**10.35**
Edu_Status (Undergraduate)	0.64	0.08	5.43
**Parents Annual Income (<RM100,000)**	**3.82**	**1.58**	**9.27**
Parents Annual Income (>RM300,000)	1.85	0.82	4.19
Sex (Female)	1.26	0.65	2.46

Significant findings are in bold.

**Table 3:** Association between never smoker and smoker with sociodemographic factors and personality traits.

Subject Characteristics	^#^ Never-Smoker, (n = 420)	Single Tobacco Users, (n = 31)	Multiple Tobacco Users, (n = 17)	* *p*-Value	χ^2^	^$^ OR	95% CI	
							Lower	Upper
Personality Traits (±SD)					-	-	-	-
Openness	26.47	24.10	26.29	0.08				
Conscientiousness	24.43	24.16	1.12	0.06				
**Extraversion**	**22.56**	**21.48**	**19.24**	**0.02**				
**Agreeableness**	**21.03**	**22.90**	**20.12**	**0.02**				
Neuroticism	22.96	22.68	22.18	0.69				
Sex (%)					0.57			
**Male**	**117 (72.1)**	**13 (41.9)**	**7 (41.2)**	**0.03**		**0.24**	**0.068**	**0.866**
**Female**	**303 (27.9)**	**18 (58.1)**	**10 (58.8)**	**0.004**		**0.19**	**0.063**	**0.601**
Parents’ Annual Income (%)					11.81			
<RM100,000	172 (41)	8 (25.8)	2 (11.8)	0.05		0.209	0.043	1.021
RM100,000–RM300,000	182 (43.3)	11 (35.5)	9 (52.9)	0.44		1.565	0.503	4.872
>RM300,000	66 (15.7)	12 (38.7)	6 (52.9)					
Ethnicity (%)					22.85			
Malay	25 (6)	2 (6.5)	0	1.0		0.00	0.000	0
**Chinese**	**297 (70.7)**	**13 (41.9)**	**5 (29)**	**0.001**		**0.109**	**0.029**	**0.411**
Indian	41 (9.8)	4 (12.9)	5 (29)	0.413		0.581	0.158	2.137
Others	57 (13.6)	12 (38.7)	7 (41)					

Data are presented as mean (±SD) for personality traits score and as a frequency and percentage for categorical variables. * Association between smoking status and personality traits score used MANOVA and multinomial logistic regression modelling was used for association between smoking status and sociodemographic factors. # This parameter is set as the reference category for multinomial logistic regression modelling. $ Measure of association for multiple tobacco users. Significant findings are in bold.

**Table 4:** Comparison of subject characteristics between tobacco product type users and non-smoking tobacco product type users.

Subject Characteristics	Non-Cigarette Smoker (n = 446)	Cigarette User (n = 22)	*p*-Value *	Non-E-Cigarette User (n = 438)	E-Cigarette User (n = 30)	*p*-Value *	Non-Water Pipe User (n = 449)	Water Pipe User (n = 19)	*p*-Value *
Age (±SD)	21.0 (2.8)	21.7 (2.8)	0.16	21.03 (2.9)	21 (2.1)	0.48	21 (2.8)	21.7 (2.8)	0.18
Personality Traits (±SD)									
Openness	26.4 (5.1)	24.8 (4.2)	0.07	26.4 (5.1)	25.1 (4.1)	0.09	26.3 (5.1)	26.3 (3.9)	0.5
Conscientiousness	24.4 (4.7)	26.5 (6.2)	**0.03**	24.4 (4.8)	25.9 (5.3)	0.06	24.5 (4.8)	24.4 (4.1)	0.47
Extraversion	22.4 (4.6)	21.0 (5.1)	0.08	22.5 (4.6)	20.2 (4.7)	**0.003**	22.5 (4.6)	19 (3.9)	**<0.001**
Agreeableness	21.1 (4.2)	21.7 (5.7)	0.25	21.1 (4.2)	21.1 (5.3)	0.49	21.1 (4.3)	20.5 (4.8)	0.27
Neuroticism	23.0 (4.8)	21.7 (5.2)	0.12	23.0 (4.8)	22.0 (5.1)	0.15	22.9 (4.8)	23.8 (3.0)	0.21
Sex (%)									
Male	125 (28.3)	12 (54.5)	**0.008**	124 (28.3)	13 (43.3)	0.08	133 (29.6)	4 (21.1)	0.42
Female	321 (72.0)	10 (45.5)		314 (71.7)	17 (56.7)		316 (70.4)	15 (78.9)	
Parents’ Annual Income (%)									
<RM100,000	178 (40)	4 (18.2)	**0.031**	177 (40.4)	5 (16.7)	**0.005**	179 (39.9)	3 (15.8)	**0.03**
RM100,000–RM300,000	192 (43)	10 (45.5)		188 (42.9)	14 (46.7)		193 (43)	9 (47.4)	
>RM300,000	76 (17)	8 (36.4)		73 (16.7)	11 (36.7)		77 (17.1)	7 (36.8)	
Ethnicity (%)									
Malay	26 (5.8)	1 (4.5)	**0.005**	26 (5.9)	1 (3.3)	**0.01**	27 (6)	0	**<0.001**
Chinese	307 (68.8)	8 (36.4)		302 (68.9)	13 (43.3)		309 (68.8)	6 (31.6)	
Indian	44 (10)	6 (27.3)		44 (10)	6 (20)		47 (10.5)	3 (15.8)	
Others	69 (15.5)	7 (31.8)		66 (15.1)	10 (33.3)		66 (14.7)	10 (52.6)	

Data are presented as mean (±SD) for continuous (age and personality traits score) variables and as a frequency and percentage for categorical variables. * Comparisons between smoking status groups used a 2-sided independent *t*-test for continuous variables and a χ^2^ test between categorical variables. Significant findings appear in bold.

**Table 5:** Correlation matrix between the personality constructs among the study participants. Numbers 1–5 of the Pearson’s correlation matrix represent variables as shown in the table’s first column.

Psychological Constructs	Mean (±SD)	Pearson Correlations				
		1	2	3	4	5
1. Openness	26.31 (5.02)	1	0.10 *	0.34 **	0.15 **	−0.11 *
2. Conscientiousness	24.51 (4.82)	0.72 *	1	0.41 *	0.53 *	0.49 *
3. Extraversion	22.37 (4.60)	0.52 *	0.41 *	1	0.25 *	0.26 *
4. Agreeableness	21.12 (4.32)	0.60 *	0.53 *	0.25 *	1	0.54 *
5. Neuroticism	22.91 (4.82)	0.52 *	0.49 *	0.26 *	0.54 *	1

* Correlation is significant at the 0.05 level. ** Correlation is significant at the 0.01 level.
